# Dexmedetomidine as an Adjuvant to Nerve Block for Cancer Surgery: A Systematic Review and Meta-Analysis

**DOI:** 10.3390/jcm13113166

**Published:** 2024-05-28

**Authors:** Christrijogo Soemartono Waloejo, Dian Anggraini Permatasari Musalim, David Setyo Budi, Nando Reza Pratama, Soni Sunarso Sulistiawan, Citrawati Dyah Kencono Wungu

**Affiliations:** 1 Department of Anesthesiology and Reanimation, Faculty of Medicine, Universitas Airlangga, Surabaya 60115, Indonesia; christrijogo@fk.unair.ac.id (C.S.W.);; 2 Faculty of Medicine, Universitas Airlangga, Surabaya 60115, Indonesia; 3 Nuffield Department of Medicine, University of Oxford, Oxford OX1 2JD, UK; 4 Institute of Tropical Disease, Universitas Airlangga, Surabaya 60115, Indonesia; 5 Department of Physiology and Medical Biochemistry, Faculty of Medicine, Universitas Airlangga, Surabaya 60115, Indonesia

**Keywords:** cancer, dexmedetomidine, PONV, regional anesthesia, meta-analysis

## Abstract

**Background/Objectives**: Our understanding of dexmedetomidine, as an adjuvant to nerve blocks in cancer surgery, is characterized by a current lack of compelling evidence, and it remains unknown whether the potential benefits of use outweigh the risks. The aim of the study was to evaluate the benefit and safety profiles of dexmedetomidine as an adjuvant to nerve blocks in cancer surgery. **Methods**: Systematic searches were conducted in MEDLINE, ScienceDirect, Cochrane Library, Springer, medRxiv, and Scopus up to 17 May 2024. Risk ratios (RR) for binary outcomes and standardized mean differences (SMDs) for continuous outcomes were quantified. **Results**: Twenty studies were identified. In breast cancer surgery, the use of dexmedetomidine reduced 24 h total morphine consumption (SMD = −1.99 [95% CI −3.01 to −0.98], *p* = 0.0001, I2 = 91%, random effects) and prolonged the requirement for morphine rescue analgesia (SMD = 2.98 [95% CI 0.01 to 5.95], *p* = 0.05, I2 = 98%, random effects). In abdominal cancer surgery, the dexmedetomidine group had lower total sufentanil consumption (SMD = −1.34 [95% CI −2.29 to −0.40], *p* = 0.005, I2 = 84%, random effects). Dexmedetomidine reduced the VAS score and decreased postoperative nausea and vomiting (PONV). No studies using dexmedetomidine reported serious adverse events. **Conclusions**: Using dexmedetomidine as an adjuvant to nerve blocks in cancer surgery could lower the VAS pain score and prolong the regional anesthesia duration, which would lead to a decrease in total opioid consumption and possibly contribute to fewer PONV events. Furthermore, the reports of no serious adverse events indicate its good safety profile.

## 1. Introduction

Surgery is one of the main treatments for cancer [1]. However, despite the apparent complete surgical removal of a tumor, some residual tissue may remain. During surgery, some cancer cells can be released into the bloodstream, leading to the tumor spreading to other organs [2]. Surgical stress can activate both the hypothalamic–pituitary–adrenal axis and the sympathetic nervous system. These systems, in turn, influence the immune response, leading to a further decrease in cell immunity. This suppression increases the likelihood of metastatic recurrence. General anesthesia is often used as the main anesthesia for cancer surgery. Some volatile anesthetic agents, like isoflurane and sevoflurane, increase several prometastatic factor transcriptions, enhancing the proliferation of tumor cells [3]. In addition to volatile anesthetic agents, opioids also directly influence tumor growth by activating transcription factors. Furthermore, as immunomodulators, opioids have the potential to increase the risk of cancer recurrence [4].

Regional anesthesia effectively lowers the neuroendocrine stress response to surgery by controlling pain or blocking sympathetic activity, decreasing catecholamine levels, and minimizing immunosuppression [3,5]. Other mechanisms of regional anesthesia that appear to provide a protective effect against tumor growth and metastasis include direct cytotoxicity; the activation of the apoptotic pathway; the inhibition of tumor cell proliferation, migration, and invasion; the modulation of gene expression through DNA demethylation; and an increase in the number of T-helper (Th) cells while maintaining the ratio of Th1 to Th2 cells [3,6]. Regional anesthesia also improves the mobilization time, shortens the time to discharge, and reduces the total opioid dose and level of volatile agents, which inhibits cancer recurrence [7].

In regional anesthesia, the length of analgesic duration is important. One way to prolong the duration of nerve blocks is the addition of adjuvants. Dexmedetomidine is an agonist of the α-2 adrenoceptor, with some anxiolytic, sympatholytic, sedative, and analgesic effects [8]. Although it has a weak analgesic effect, dexmedetomidine can be used as a helpful analgesic adjuvant. Multiple pathways, including spinal, supraspinal, ganglionic, and peripheral effects, are responsible for the dexmedetomidine analgesic pathway [8,9]. Furthermore, due to its antiemetic properties, dexmedetomidine is associated with a decreased incidence of postoperative nausea and vomiting (PONV) [10], suggesting its potential benefit in postoperative cancer surgery.

Several randomized controlled trials (RCTs) on the use of dexmedetomidine as an adjuvant to nerve blocks for cancer surgery have recently been published. However, at the time of this study being written, the application of dexmedetomidine as an adjuvant to nerve blocks in cancer surgery remains debatable due to conflicting results, demanding further investigation. Therefore, in this systematic review and meta-analysis, we aim to comprehensively evaluate the benefit and safety profiles of dexmedetomidine as an adjuvant to nerve blocks in cancer surgery.

## 2. Materials and Methods

This meta-analysis followed the 2020 Preferred Reporting Items for Systematic Review and Meta-analysis (PRISMA) guideline [11], and was registered in the PROSPERO database with a registration number of CRD42023460288.

### 2.1. Eligibility Criteria

We included a randomized controlled trial (RCT) in this systematic review and meta-analysis. The titles and abstracts of all retrieved studies were screened based on the following eligibility criteria: (1) studies considering patients with cancer surgery; (2) patients receiving dexmedetomidine as an adjuvant in truncal regional nerve blocks; (3) research with randomized controlled trial study designs; (4) studies available in the English language; and (5) eligible studies reporting at least one of our outcomes of interest. Our outcomes included clinical outcomes, intraoperative outcomes, postoperative pain scores, laboratory outcomes and adverse events. Review articles, irrelevant studies, non-human studies, and duplicates were excluded.

### 2.2. Search Strategy and Selection of Studies

**Figure 1:**
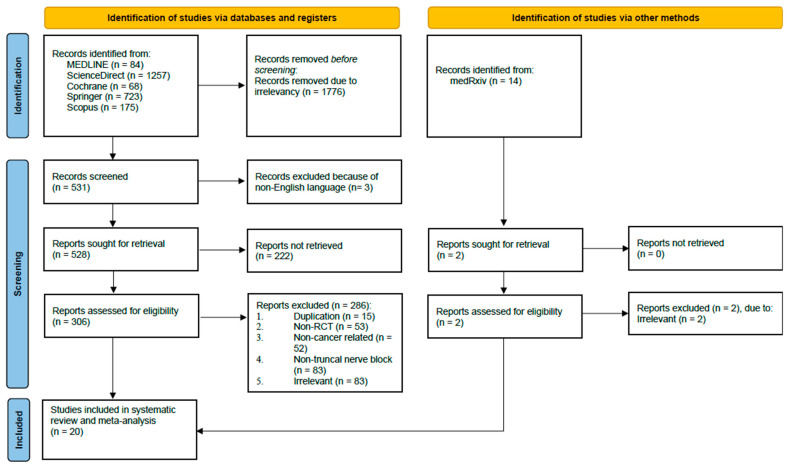
PRISMA flow diagram of study selection process.

DAPM and DSB performed a systematic literature search in MEDLINE, ScienceDirect, Cochrane Library, Springer, and Scopus on 17 May 2024. Manual searches (e.g., in medRxiv) and bibliographical searches were also conducted to obtain additional evidence. The following keywords were used: “((Regional anaesthesia) OR (Nerve block) OR (Truncal nerve block)) AND (Dexmedetomidine) AND ((Cancer) OR (Cancer surgery))”. Additional details about the search strategy can be found in Supplementary Materials. Any disagreements were resolved among all authors by discussion until a consensus was reached. The details of study selection were documented in a PRISMA flow diagram (Figure 1).

### 2.3. Data Extraction

Relevant data were extracted from each selected study using structured and standardized forms. The following data were extracted: first author’s name and publication year, study design, country, surgery types, sample size, patient’s age, ASA physical status, clinical outcomes, intraoperative outcomes, postoperative pain scores, laboratory outcomes, and adverse events.

### 2.4. Quality Assessment

The quality of each study was assessed independently by two authors (DSB and DAPM) using the Cochrane risk-of-bias tool for randomized trials (RoB 2) which is accessible at https://sites.google.com/site/riskofbiastool/welcome/rob-2-0-tool and accessed on 17 May 2024. Any discrepancies were resolved by discussion until a consensus was reached. The Grading of Recommendation Assessment, Development, and Evaluation (GRADE) system was used to evaluate the quality of the evidence in the findings [12].

### 2.5. Statistical Analysis

Primary analyses were carried out using Review Manager version 5.4 (The Cochrane Collaboration). Pooled risk ratios (RRs) for dichotomous outcomes were evaluated using the Mantel–Haenszel method. Standardized mean differences (SMDs) of continuous outcomes were pooled using the inverse variance method. The heterogeneity of the study was assessed using I2 statistics. The random effects analysis was employed to estimate effect size. We used Begg’s funnel plots to perform publication bias analysis. If present, the trim-and-fill method was used. All results of statistical analysis with a *p*-value ≤ 0.05 were considered statistically significant. Leave-one-out sensitivity analysis was conducted to find the source of statistical heterogeneity and demonstrate how each study affected the overall result.

## 3. Results

### 3.1. Study Selection

This study obtained 2307 and 14 records from database and manual searches, respectively. After screening the titles and abstracts, 306 potential articles were selected for full-text screening. Following this, 20 studies were included. This study selection process is summarized in the PRISMA flow chart (Figure 1). According to Cochrane’s Risk of Bias 2 (RoB2) assessment, seventeen RCTs [13,14,15,16,17,18,19,20,21,22,23,24,25,26,27,28,29] were considered to be low-risk and three RCTs [30,31,32] had some concerns. Moreover, the quality of the included studies is summarized in Supplementary Materials (Table S2).

### 3.2. Study Characteristics

The characteristics and outcomes of the 20 included studies, with a total of 617 patients receiving truncal regional anesthesia combined with dexmedetomidine and 616 subjects in the control group without dexmedetomidine, are shown in Table 1 and Table 2. Eleven studies were from Egypt, six studies were from China, and three studies were from India. Surgery was found to be most commonly performed for breast cancer in our meta-analysis, followed by abdominal cancer, thoracic cancer, and radical cystectomy. The dexmedetomidine dosage ranged from 0.5 μg/kg to 2 μg/kg. The summary findings of this review are shown in Table 3, and the certainty of evidence of findings reported using the GRADE system is presented in the Supplementary Materials (Table S3).

### 3.3. Clinical Outcomes

**Figure 2:**
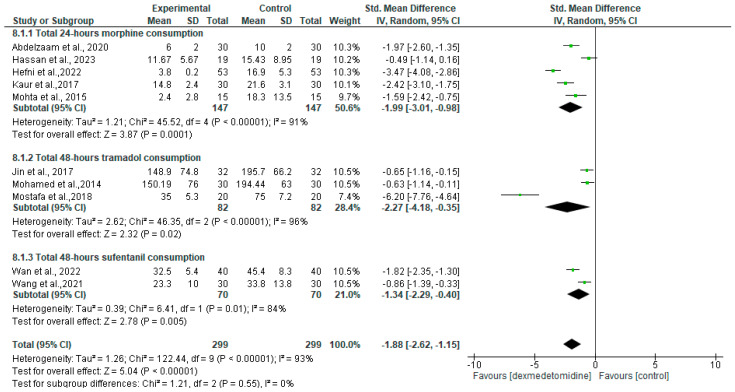
Forest plot of meta-analysis for total opioid consumption. SD, standard deviation; IV, inverse variance; CI, confidence interval [13,16,17,18,20,21,22,23,26,31].

Five studies into breast cancer surgery reported on 24 h postoperative morphine consumption [13,16,18,21,31]. The meta-analysis of those studies concluded that total 24 h morphine consumption in the dexmedetomidine group was lower than that of the control group (SMD = −1.99 [95% CI −3.01 to −0.98], *p* = 0.0001, I2 = 91%, random effects) (Figure 2). Three studies reported on 48 h postoperative tramadol consumption following breast cancer surgery [17,20,22]. As shown in Figure 2, dexmedetomidine treatment significantly reduced tramadol consumption (SMD = −2.27 [95% CI −4.18 to −0.35], *p* = 0.02, I2 = 96%, random effects). The dexmedetomidine group also had a lower dose of flurbiprofen, an nonsteroidal anti-inflammatory drug (NSAID), compared to that without the dexmedetomidine adjuvant (100 [IQR: 52–115] vs. 150 [IQR: 94–160], *p* = 0.038) [23].

Two out of three abdominal cancer surgery reported on 48 h postoperative sufentanil consumption [26,27]. The meta-analysis of those studies concluded that sufentanil consumption was significantly lower in the dexmedetomidine group (SMD = −1.34 [95% CI −2.29 to −0.40], *p* = 0.005, I2 = 84%, random effects) (Figure 2). Similar to the breast cancer and abdominal surgery, in thoracic cancer [28] and radical cystectomy [32], dexmedetomidine adjuvant use decreased the need for analgesic consumption.

**Figure 3:**
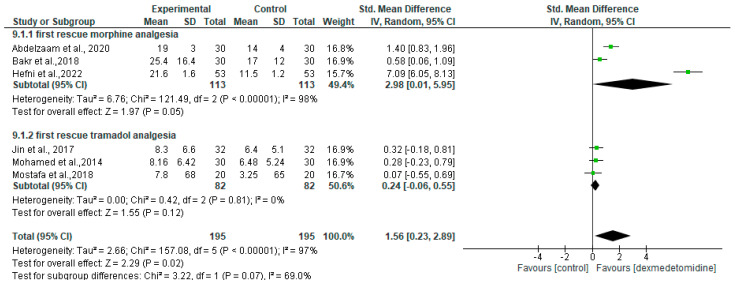
Forest plot of meta-analysis for time to first rescue analgesia. SD, standard deviation; IV, inverse variance; CI, confidence interval [13,14,16,17,20,22].

In breast cancer surgery, the time until the first use of rescue morphine analgesia was mentioned in three RCTs [13,14,16]. The meta-analysis showed that dexmedetomidine treatment prolonged the requirement for morphine rescue analgesia (SMD = 2.98 [95% CI 0.01 to 5.95], *p* = 0.05, I2 = 98%, random effects). However, the first request for tramadol in the dexmedetomidine group during breast cancer surgery was insignificant (SMD = 0.24 [95% CI −0.06 to 0.55], *p* = 0.12, I2 = 0%, random effects) [17,20,22] (Figure 3). Although it was not statistically significant, a higher percentage of patients in the erector spinae plane block (ESPB) group without dexmedetomidine required rescue analgesia in the first 24 h following thoracic cancer surgery (71.4% vs. 52.4%, *p* = 0.204) [28].

Making comparisons to the control group, dexmedetomidine use considerably improved the time to discharge (*p* = 0.021) and allowed for earlier mobilization (*p* < 0.001) in breast cancer surgery [21]. Patient satisfaction ratings were published in three studies [19,24,29]. In comparison to the ropivacaine-only group, the overall QoR-15 scores were considerably higher in the group treated with deep serratus anterior plane block (dSAPB) with ropivacaine plus dexmedetomidine 24 h post modified radical mastectomy, indicating a better recovery, *p* = 0.016 [24]. Following lung cancer surgery, twenty out of thirty patients (66.7%) in the dexmedetomidine group scored five points on a 5-point Likert scale, and this difference was significant when compared to the control group (*p* < 0.001) [29].

### 3.4. Intraoperative Outcome

Four studies reported on intraoperative outcomes, including intraoperative hemodynamic parameters and the onset of peripheral nerve blocks [17,19,20,22]. Regarding the hemodynamic parameters measured during the intraoperative period, there were consistent results between the research of Mohamed et al., 2014 [20] and Jin et al., 2017 [17], where there was a significant reduction in heart rate starting at 30 min in the dexmedetomidine group compared to the control group (69.8 ± 8.29 vs. 79.33 ± 6.62, *p* < 0.001 and 68.4 ± 8.3 vs. 79.1 ± 6.6, *p* < 0.05), respectively. Nevertheless, at 60 min into the intraoperative period, the mean heart rate in the dexmedetomidine group gradually increased. This was consistently reported in both trials, but was not significantly different from the control group (74.43 ± 11.03 vs. 80.40 ± 8.45, *p* > 0.05 and 74.6 ± 10.7 vs. 80.1 ± 8.4, *p* > 0.05). Meanwhile, at 90 min (75 ± 6 vs. 77 ± 5, *p* = 0.07) [22] and 120 min (77.8 ± 9.8 vs. 80.3 ± 8.5, *p* > 0.05) [17], there were no significant heart rate differences between the dexmedetomidine and control groups. Lakshmi et al. 2022 [19] reported that the mean heart rates in the dexmedetomidine and control groups, examined intraoperatively, are not statistically significant (79.90 ± 5.89 vs. 80.73 ± 6.79, *p* = 0.4). The intraoperative systolic blood pressure showed a significant reduction at 30 min in dexmedetomidine groups (101.33 ± 11.67 vs. 130.00 ± 11.49, *p* < 0.001), but then returned to baseline levels at 120 min (127.20 ± 12.79 vs. 130.00 ± 11.49, *p* > 0.05) [20]. Changes in intraoperative systolic blood pressure (30 min: 102.4 ± 11.8 vs. 129.1 ± 11.6, *p* < 0.05; 120 min: 127.0 ± 12.5 vs. 129.1 ± 11.6, *p* > 0.05) and diastolic blood pressure (30 min: 64.9 ± 7.8 vs. 81.4 ± 7.5, *p* < 0.05; 120 min: 78.0 ± 7.0 vs. 81.4 ± 7.5, *p* > 0.05) in the dexmedetomidine group were similar to those relating to heart rate, where a significant drop occurred at 30 min and then became stable until 120 min compared to baseline [17]. Moreover, the average time needed until the start of sensory block was longer in the control group than in the dexmedetomidine group and was statistically significant (4.76 ± 0.707 min vs. 3.4 ± 0.70 min; *p* = 0.0001) [19].

### 3.5. Postoperative Mean Pain Score

**Figure 4:**
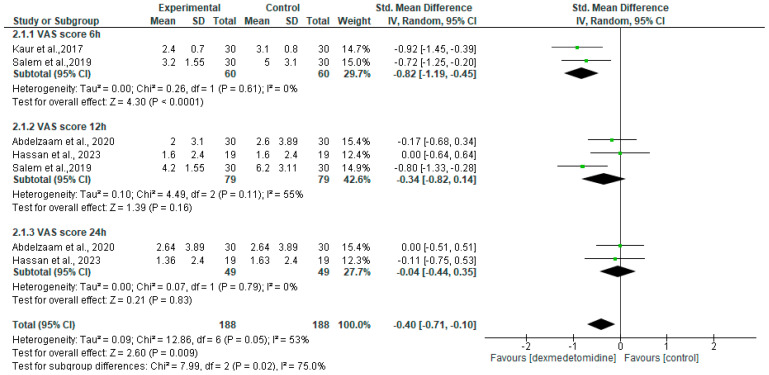
Forest plot of meta-analysis for VAS score. SD, standard deviation; IV, inverse variance; CI, confidence interval [13,18,25,31].

We included four RCTs, which used morphine as analgesic and reported VAS at 6 h, 12 h, or 24 h postoperative, in our meta-analysis [13,18,25,31]. Overall, the dexmedetomidine group had a VAS lower than the control group (SMD = −0.40 (95% CI −0.71 to −0.10), *p* = 0.009, I2 = 53%, random effects) (Figure 4). Although this was significant statistically, it may not be clinically meaningful. The use of a dexmedetomidine adjuvant significantly lowered the rest VAS score at 12 h and 16 h after surgery, with *p* < 0.001 and *p* = 0.013 respectively. However, there were no significant changes at the postanesthesia care unit (PACU), 4, 8, 20, and 24 h after surgery [13]. Deep-SAPB combined with dexmedetomidine significantly reduced the acute VAS score at rest at 12 h and exercise at 12 and 24 h after surgery (both *p* < 0.05). There was no significant difference between resting (*p* = 0.125) and exercise (*p* = 0.104) at 48 h after surgery [24].

### 3.6. Adverse Events

**Figure 5:**
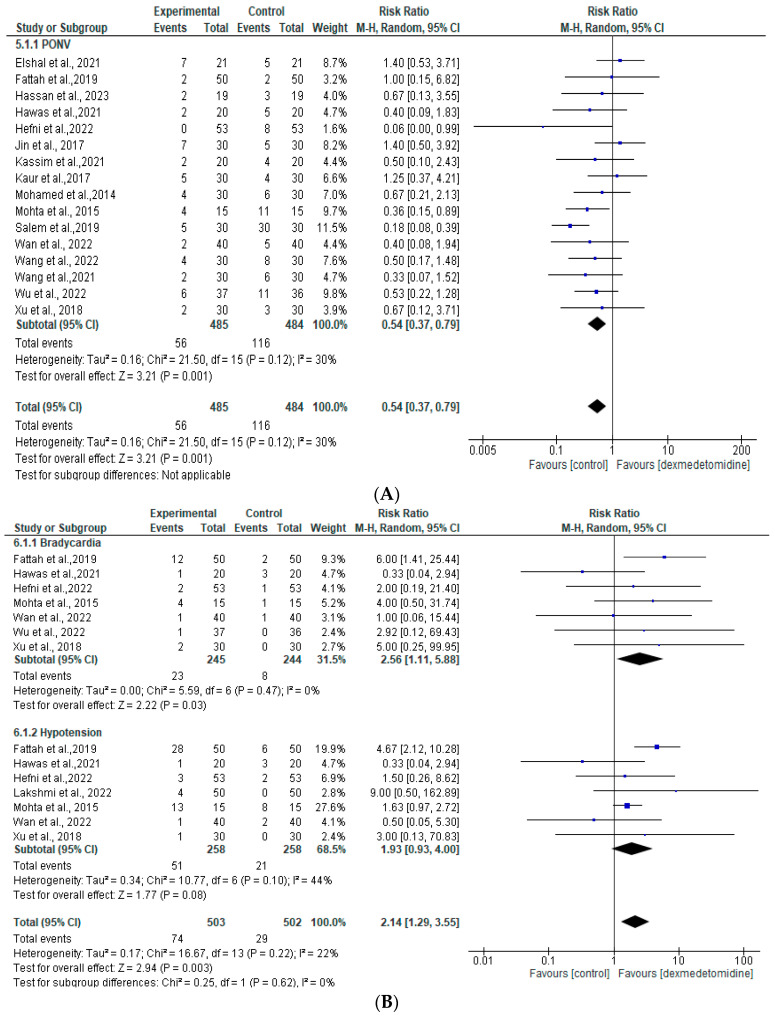
Forest plot of meta-analysis for any adverse events: (**A**) PONV; (**B**) bradycardia and hypotension [15,16,17,18,19,20,21,23,24,25,26,27,28,29,30,31,32].

There were seventeen studies that reported about the adverse events of dexmedetomidine adjuvant in peripheral nerve blocks [15,16,17,18,19,20,21,23,24,25,26,27,28,29,30,31,32]. Following cancer surgery, bradycardia, hypotension, nausea, and vomiting were the most frequently reported adverse effects. Based on our meta-analysis, dexmedetomidine was able to reduce PONV compared to the control group (RR 0.54 [95% CI 0.37 to 0.79], *p* = 0.001, I2 = 30%, random effects) (Figure 5A). The incidence of bradycardia was significant in the group which received dexmedetomidine (RR 2.56 [95% CI 1.11 to 5.88], *p* = 0.03, I2 = 0%, random effects). However, hypotension did not significantly differ between the dexmedetomidine and control groups (RR 1.93 [95% CI 0.93 to 4.00], *p* = 0.08, I2 = 44%, random effects) (Figure 5B). No studies using dexmedetomidine reported serious adverse events, such as respiratory depression, cardiovascular problems, or death related to adverse events. The funnel plot did not indicate any publication bias (Figures S1–S4).

## 4. Discussion

Studies on the use of adjuvants to maximize the duration of analgesia during regional anesthesia are interesting because dexmedetomidine has been increasingly reported as one of the potential agents that could improve perioperative and postoperative outcomes. Since the introduction of dexmedetomidine as an adjuvant anesthesia, the usage of opioids, inhalational anesthetics, and intravenous anesthetics has decreased significantly [8]. Its opioid-sparing effect is able to reduce opioid requirements without increasing the incidence of opioid side effects, particularly respiratory depression [33]. In this meta-analysis, dexmedetomidine was shown to reduce the amount of opioids needed during various types of cancer surgery, prolong the requirement for morphine rescue analgesia, and lower the VAS score at 6 h, although this may not be clinically meaningful. Additionally, the administration of dexmedetomidine reduced PONV. Meanwhile, the incidence of hypotension and bradycardia was numerically higher in the dexmedetomidine group compared to the control group, although it did not reach the level of statistical significance for the incidence of hypotension. The mechanisms of dexmedetomidine action in peripheral nerve blocks include maintaining hyperpolarized cells by inhibiting the next action potential through the potassium channel, maintaining the depolarization of the cell, and having local action [34,35]. Dexmedetomidine prolongs nerve blocks via a number of processes, including direct action on the nerve, the attenuation of local anesthetic-induced neurotoxicity, a reduction in local blood flow, and local vasoconstriction at the spinal and supraspinal levels [34,36,37].

Dexmedetomidine, as an adjuvant to local analgesia, can reduce the inflammation and perineural damage caused by local anesthetics [37]. In transabdominal plane (TAP) blocks, adjuvant dexmedetomidine is often distributed systemically and has direct central effects on the locus coeruleus [38]. Another central action of dexmedetomidine is the inhibition of substance-*p* release into the nociceptive pathway [21]. During gastric cancer surgery, the group receiving intravenous dexmedetomidine uses less propofol and remifentanil than the control group [39]. In addition to cancer surgery, the continuous infusion of dexmedetomidine has been demonstrated to decrease the overall opioid use in orthopedic surgery compared to the midazolam group. The group administered with dexmedetomidine would receive 62.06% fewer opioids than the group administered with midazolam [36]. Opioids, however, are able to induce hyperalgesia, which increases pain and opioid consumption. An option for opioid-induced hyperalgesia treatment might be the adjuvant dexmedetomidine [40]. Dexmedetomidine has also been shown to reduce the need for various analgesics, such as paracetamol [41,42]; flurbiprofen [18]; and ketorolac [43].

The adjuvant dexmedetomidine can extend the duration of the anesthetic agent’s effect, resulting in a prolonged first rescue analgesic time in the dexmedetomidine group [13,14,16,17,20,22,25,32]. The analgesic duration of dexmedetomidine injected locally, compared to intravenous dexmedetomidine administration in mid-forearm blocks, is significantly longer (997 ± 243 min vs. 654 ± 159 min, respectively) [35]. Perineural dexmedetomidine administration can prolong the duration of analgesia because of the absorption and redistribution of the perineural dexmedetomidine, triggering systemic effects [44]. At the peripheral level, by activating α2 adrenoceptors in peripheral blood vessels, this causes vasoconstriction, delaying the absorption of local anesthetics and lengthening their block time [27]. With 0.75% ropivacaine, the perineural injection of dexmedetomidine can prolong ultrasound-guided ulnar nerve blocks by approximately 60%, as compared to 10% when administered systemically [45]. In another study on lobectomy surgery, by mixing 0.5% ropivacaine with 1 µg/kg of perineural dexmedetomidine, ESPB was prolonged by about 120% [46]. When administered around the nerve, dexmedetomidine enhanced the cation channel, which prevented cell depolarization. Consequently, dexmedetomidine combined with regional anesthesia can improve nerve conduction and have more potent analgesic effects than the use of local anesthetics alone [24].

In our study, dexmedetomidine was able to shorten the time to discharge [21]. An epidural block with dexmedetomidine could potentially reduce the duration of hospitalization following colorectal cancer surgery (7.6 ± 2.0 vs. 10.3 ± 1.8 days, *p* < 0.001) [47]. Similar to the findings of Ke et al., 2023 [33], patients in the opioid group had significantly longer postoperative hospital stays and total hospital stays than the dexmedetomidine group. These effects might be attributed to opioid-related side effects postoperatively. The study showed that dexmedetomidine did not require rescue analgesia within two hours of the procedure, and 88% patients undergoing modified radical mastectomy or breast conservative surgery could be discharged on the same day [48]. The use of dexmedetomidine as an adjuvant in ESPB may also reduce postoperative hospital stays compared to adjuvant dexamethasone administration. The combination of ropivacaine and dexmedetomidine extend the sensory block to 18 h. Patients who receive an adequate analgesic report better comfort, earlier mobilization, and a lower risk of pulmonary problems, all of which result in shorter hospital stays [46]. Another study showed that there were no differences in terms of the length of hospital stays in the dexmedetomidine group [49].

In addition, patient satisfaction is higher in the dexmedetomidine group [19,24,29]. The findings further show that, during day-care breast cancer surgery, the dexmedetomidine group had higher overall patient satisfaction than the normal saline group (*p* < 0.0001). Fewer side effects and early ambulation could be the cause of this [48]. The dexmedetomidine group has an mini mental state examination (MMSE) score higher than that of the control group [47]. Dexmedetomidine may have a protective effect on the incidence of postoperative delirium and POCD (postoperative cognitive dysfunction) [47,50]. There is an association between POCD pathogenesis and the inflammatory response. Intraoperative dexmedetomidine reduces inflammation because it substantially decreases the level of proinflammatory cytokiness [51]. The improved patient satisfaction with the dexmedetomidine adjuvant could be attributed to these effects.

The use of adjuvant dexmedetomidine could reduce the pain score after surgery [13,14,17,18,20,23,24,25,30,31,32,37,47]. We found significance in the statistic [(SMD = −0.40 (95% CI −0.71 to −0.10), *p* = 0.009)], but it may not be clinically meaningful due to the fact that the numbers are too small. Dworkin et al. [52] stated that individual pain intensity scores should decrease by 1.0 points to represent “minimal” or “little” change, and decrease by 2.0 to 2.7 points to be more clinically significant for patients. Within major abdominal cancer surgery, an epidural infusion of dexmedetomidine considerably reduces pain intensity within the first 48 h following surgery [9]. The use of 1 µg/kg of dexmedetomidine as an adjuvant in ESPB significantly decreases VAS score in the PACU and 2, 4, 12, and 24 h after surgery [46]. The opioid-free group, which used dexmedetomidine in lobectomy surgery, had a lower postoperative VAS score at 0, 6, 12, and 24 h [53]. Most studies found potential benefits in terms of acute pain scores. Another study found that the group receiving dexmedetomidine had a decreased brief pain inventory (BPI), a measure of chronic pain severity, at three months following the mastectomy. Additionally, those in the dexmedetomidine groups had a higher quality of life [41].

During the first half-hour of intraoperative hemodynamics, dexmedetomidine produces an average heart rate and blood pressure, with levels lower than those in the control; nevertheless, both of these parameters will subsequently return to baseline. This is because dexmedetomidine takes around 15 min to start acting, and it peaks after an hour. For adults, the distribution half-life (t½α) of dexmedetomidine is 6 min at doses ranging from 0.2 to 0.7 μg/kg/jam, while the elimination half-life (t½β) is around 2.0 to 2.5 h and the clearance is 39 L/min. A stable plasma concentration may be achieved by both adults and children with the same infusion rate [54,55]. These dexmedetomidine properties will prevent surgery stress responses by decreasing blood pressure and heart rate [56].

Our results show that hypotension and bradycardia events are increasing in the dexmedetomidine group. This suggests the dexmedetomidine adjuvant should be monitored carefully. In addition, dexmedetomidine use could decrease PONV incidence compared to the control group. These benefits could be due to the antiemetic effect related to α2 agonist, but as of now, the connection remains unclear [57]. The antiemetic effect of dexmedetomidine may be explained by decreased sympathetic activity. Furthermore, elevated blood levels of catecholamine might induce nausea and vomiting. The higher total opioid dose in the control group was attributed to an increase in PONV incidence. Opioid use was associated with the PONV incidence in many studies [58,59]. POhNV could be minimized by adding dexmedetomidine as an adjuvant, which also decreases opioid use.

Finally, this meta-analysis had several limitations. First, the included RCTs had a low number of participants and three out of twenty-five RCTs were open-label [30,31,32]. The open-label design had the potential to lead to a higher selection bias and a lower quality of evidence. Second, the inclusion of studies from different clinical settings complicated the results. Third, the different surgical types and length of operation contributed to the heterogeneity in opioid consumption and efficacy of dexmedetomidine. Fourth, there were limited data regarding cancer outcomes. Lastly, further well-powered studies with the more extensive adjustment of confounders, as well as larger double-blind RCTs, are warranted to address some limitations of our current meta-analysis.

## 5. Conclusions

Overall, our systematic review and meta-analysis highlighted the observed benefits of using dexmedetomidine as an adjuvant in truncal nerve blocks for cancer surgery, including reduced total analgesic consumption, prolonged nerve block duration, and a shortened time to discharge with a higher patient satisfaction score. Meanwhile, postoperatively, dexmedetomidine may lower the VAS pain score and the PONV incidence. Nevertheless, this study found that hypotension and bradycardia incidence were numerically higher in the dexmedetomidine group compared to the control group. Although it did not reach statistical significance for the incidence of hypotension, careful monitoring is warranted.

## Figures and Tables

**Table 1:** Characteristics of the included studies.

Reference	Study Design	Country	Surgery	Sample Size		Age (y)		ASA Physical Status		Dosage and Administration	Comparison
						Mean ± SD or Median (IQR)					
				Intervention	Control	Intervention	Control	Intervention	Control		
								N	N		
Abdelzaam et al., 2020 [13]	RCT	Egypt	Breast cancer surgery	30	30	46 ± 6	43 ± 7	I–II	I–II	GA + serratus plane block: 0.25% bupivacaine 0.5 mL/kg + dexmedetomidine 0.5 μg/kg	GA + serratus plane block: 0.25% bupivacaine 0.5 mL/kg
Bakr et al., 2018 [14]	RCT	Egypt	Breast cancer surgery	30	30	47.3 ± 9.7	48.5 ± 13.7	I–II	I–II	GA + pecs block: 30 mL of 0.25% bupivacaine + 1 μg/kg dexmedetomidine	GA + pecs block: 30 mL of 0.25% bupivacaine
Fattah et al., 2019 [30]	RCT	Egypt	Breast cancer surgery	50	50	20–55	20–55	II–III	II–III	GA + TPVB: 20 mL of bupivacaine 0.5% + 0.5 μg/kg dexmedetomidine	GA + TPVB: 20 mL of 0.5% bupivacaine
Hassan et al., 2023 [31]	RCT	Egypt	Breast cancer surgery	19	19	45.4 ± 12.7	49.5 ± 11.9	II	II	GA + ESPB: 19 mL of bupivacaine 0.5% + 1 mL of normal saline containing 1 μg/kg dexmedetomidine	GA + ESPB: 20 mL of bupivacaine 0.5%
Hawas et al., 2021 [15]	RCT	Egypt	Breast cancer surgery	20	20	41.8 ± 2.6	45.3 ± 1.7	II	II	GA + serratus plane block: 30 mL of bupivacaine 0.25% + 1 μg/kg dexmedetomidine	GA + serratus plane block: 30 mL of bupivacaine 0.25% + 25 μg fentanyl
Hefni et al., 2022 [16]	RCT	Egypt	Breast cancer surgery	53	53	47.2 ± 5.2	46.1 ± 4.2	I–III	I–III	GA + pecs block: 30 mL of bupivacaine 0.25% + 1 μg/kg dexmedetomidine in 2 mL volume	GA + pecs block: 30 mL of bupivacaine 0.25%
Jin et al., 2017 [17]	RCT	China	Breast cancer surgery	32	32	57.6 ± 10.3	58.8 ± 11.0	I–III	I–III	GA + TPVB: 20 mL of 0.25% bupivacaine + 1 mg/kg dexmedetomidine	GA + TPVB: 20 mL of 0.25% bupivacaine
Kaur et al., 2017 [18]	RCT	India	Breast cancer surgery	30	30	51.6 ± 10	46.2 ± 10	I–II	I–II	GA + pecs block: 30 mL of 0.25% ropivacaine + dexmedetomidine 1 μg/kg	GA + pecs block: 30 mL of 0.25% ropivacaine
Lakshmi et al., 2022 [19]	RCT	India	Breast cancer surgery	50	50	50.84 ± 6.36	47.8 ± 4.94	I–III	I–III	GA + TPVB: 0.3 mL/kg of ropivacaine 0.5% + 1 μg/kg Dexmedetomidine	GA + TPVB: 0.3 mL/kg Ropivacaine 0.5% + 1 mL normal saline
Mohamed et al., 2014 [20]	RCT	Egypt	Breast cancer surgery	30	30	50.50 ± 7.7	50.36 ± 60	I–III	I–III	GA + TVPB: 20 mL of bupivacaine 0.25% + 1 μg/kg dexmedetomidine	GA + TVPB: 20 mL of bupivacaine 0.25%
Mohta et al., 2015 [21]	RCT	India	Breast cancer surgery	15	15	46.6 ± 10.5	49.9 ± 10.6	I–III	I–III	GA + PVB: 0.3 mL/kg of 0.5% bupivacaine + dexmedetomidine 1 μg/kg in a volume of 1 mL	GA + PVB: 0.3 mL/kg of 0.5% bupivacaine + 1 mL normal saline
Mostafa et al., 2018 [22]	RCT	Egypt	Breast cancer surgery	20	20	55.9 ± 6	55.8 ± 5.8	I–III	I–III	GA + PVB: 0.3 mL/kg of 0.5% bupivacaine + dexmedetomidine 1 µg/kg	GA + PVB: 0.3 mL/kg of 0.5% bupivacaine + normal saline 1 mL
Wang et al., 2021 [23]	RCT	China	Breast cancer surgery	30	30	51.93 ± 9.18	52.83 ± 8.76	I–II	I–II	GA + ESPB: 30 mL of 0.33 ropivacaine + 30 mL of dexmedetomidine 1 μg/kg	GA + ESPB: 30 mL of 0.33% ropivacaine
Wu et al., 2022 [24]	RCT	China	Breast cancer surgery	37	36	54.62 ± 7.44	54.08 ± 6.28	I–II	I–II	GA + dSAPB: 30 mL of 0.375% ropivacaine + dexmedetomidine 1 μg/kg	GA + dSAPB: 30 mL of 0.375% ropivacaine
Salem et al., 2019 [25]	RCT	Egypt	Abdominal cancer surgery	30	30	NR	NR	I–II	I–II	GA + BRSB: 20 mL of 0.25% bupivacaine + dexmedetomidine 2 μg/kg	GA + BRSB: 20 mL of 0.25% bupivacaine
Wan et al., 2022 [26]	RCT	China	Gastric cancer	40	40	58.6 ± 10.77	57.2 ± 11.34	I–III	I–III	GA + TVPB: 15 mL 0.5% of ropivacaine + 2 mL dexmedetomidine (1 μg/kg)	GA + TVPB: 15 mL of ropivacaine (0.5%) + 2 mL normal saline
Wang et al., 2022 [27]	RCT	China	Abdominal cancer surgery	30	30	57.9 ± 6.0	55.8 ± 7.0	I–III	I–III	GA + ESPB: 28 mL of 0.5% ropivacaine + interfascial dexmedetomidine 0.5 μg/kg in 2 mL	GA + ESPB: 28 mL of 0.5% ropivacaine + 2 mL of normal saline
Elshal et al., 2021 [28]	RCT	Egypt	Thoracic cancer surgey	21	21	46.76 ± 9.89	44.62 ± 10.77	II	II	GA + ESPB: 28 mL of bupivacaine 0.25% + 2 mL of dexmedetomidine 0.5 μg/kg	GA + ESPB: 28 mL of bupivacaine 0.25% + 2 mL saline
Xu et al., 2018 [29]	RCT	China	Lung cancer	30	30	59.2 ± 7 9.7	59.5 ± 7 9.7	I–II	I–II	GA + TVPB: 75 mg/20 mL of ropivacaine 0.375% + dexmedetomidine 1 μg/kg	GA + TVPB: 75 mg/20 mL of ropivacaine 0.375%
Kassim et al., 2021 [32]	RCT	Egypt	Radikal cystectomy	20	20	61.5 ± 6.8	60.4 ± 6.8	I–II	I–II	GA + TAP block: 20 mL of 0.25% bupivacaine + dexmedetomidine 1 μg/kg	GA + TAP block: 20 mL of 0.25% bupivacaine + 2 mL normal saline

Pecs: pectoral; ESPB: erector spinae plane block; TPVB: thoracic paravertebral block; PVB: paravertebral block; dSAPB: deep serratus anterior plane block; BRSB; bilateral rectus sheath block; TAP: transversus abdominis plane; GA: general anesthesia.

**Table 2:** Outcomes of individual studies.

Reference	Clinical Outcome			Intraoperative Outcome	Mean Pain Score		Laboratory Outcome		Any Adverse Events	
	N (%)			N (%)	N (%)		(Mean ± SD)		N (%)	
	Intervention	Control	Intervention	Control	Intervention	Control	Intervention	Control	Intervention	Control
Abdelzaam et al., 2020 [13]	Time of first rescue dose (h): 19 ± 3 Total morphine consumption 24 h postoperatively (mg): 6 ± 2	Time of first rescue dose (h): 14 ± 4 Total morphine consumption 24 h postoperatively (mg): 10 ± 2	NR	NR	VAS score at rest: PACU: 1 (0–4) 4 h: 1 (0–3) 8 h: 2 (0–4) 12 h: 2 (0–4) 16 h: 2 (0–5) 20 h: 3 (0–5) 24 h: 3 (0–5) VAS score at movement: PACU: 1 (0–4) 4 h: 1 (0–4) 8 h: 2 (0–4) 12 h: 2 (0–4) 16 h: 2 (0–4) 20 h: 3(0–6) 24 h: 3(0–6)	VAS score at rest: PACU: 0 (0–4) 4 h: 1 (0–4) 8 h: 2 (0–3) 12 h: 3 (0–5) 16 h: 3 (0–5) 20 h: 3 (0–5) 24 h: 3 (0–5) VAS score at movement: PACU: 0 (0–4) 4 h: 1 (0–4) 8 h: 2 (1–3) 12 h: 3(1–6) 16 h: 3(0–5) 20 h: 4(0–6) 24 h: 4(0–6)	NR	NR	NR	NR
Bakr et al., 2018 [14]	Time to first request of analgesia (h): 25.4 ± 16.4 Total PCA morphine 48 h postoperatively (mg): 9 ± 3.6	Time to first request of analgesia (h): 17 ± 12 Total PCA morphine 48 h postoperatively (mg): 12 ± 3.6	NR	NR	VAS score 12 h: 2.1 ± 1	VAS score 12 h: 2.7 ± 1.1	Cortisol level (μg/dL): 205.9 ± 142.6 prolactin level (ng/mL): 28.3 ± 22.1	Cortisol level (μg/dL): 257.3 ± 163.2 prolactin level (ng/mL): 41.7 ± 21.2	NR	NR
Fattah et al., 2019 [30]	Total opioid consumption 24 h postoperatively (mg): 5 Need for extra sedation: 27 (61.4)	Total opioid consumption 24 h postoperatively (mg): 5.15 Need for extra sedation: 28 (63.6)	NR	NR	VAS score 30 min: 1.5 ± 1	VAS score 30 min: 1 ± 1.75	NR	NR	Nausea and vomiting: 2 (4.5%) Bradycardia: 12 (27.3%) Hypotension: 28 (63.6%)	Nausea and vomiting: 2 (4.5%) Bradycardia: 2 (4.5%) Hypotension: 6 (13.6%)
Hassan et al., 2023 [31]	Total postoperative morphine consumption 24 h postoperatively (mg): 13.0 (6.0–32.0)	Total postoperative morphine consumption 24 h postoperatively (mg): 11.0 (4.0–22.0)	Intraoperative fentanyl consumption (μg): 110 (60.0−210.0)	Intraoperative fentanyl consumption (μg): 120.0 (60.0−200.0)	Postoperative numeric rating score at rest: After 30 min: 2 (1–3) After 2 h: 2 (0–3) After 4 h: 2 (0–3) After 8 h: 2 (0–3) After 12 h: 2 (0–3) After 24 h: 1 (0–3) Postoperative numeric rating score on movement: After 30 min: 3 (2–6) After 2 h: 3 (1–5) After 4 h: 3 (1–5) After 8 h: 3 (1–6) After 12 h: 3 (1–6) After 24 h: 2 (1–4)	Postoperative numeric rating score at rest After 30 min: 2 (1–3) After 2 h: 2 (0–3) After 4 h: 2 (0–3) After 8 h: 2 (0–3) After 12 h: 2 (0–3) After 24 h: 2 (0–3) Postoperative numeric rating score on movement: After 30 min: 3 (1–4) After 2 h: 4 (2–6) After 4 h: 3 (2–4) After 8 h: 3 (2–6) After 12 h: 3 (2–6) After 24 h: 2 (1–4)	NR	NR	Nausea and vomiting (PONV): 2 (10.5%) Pruritis: 0 (0) Respiratory depression: 0 (0) Block-related complication: 0 (0)	Nausea and vomiting (PONV): 3 (15.8%) Pruritis: 0 (0) Respiratory depression: 0 (0) Block-related complication: 0 (0)
Hawas et al., 2021 [15]	Total pethidine 24 h postoperatively (mg): 61 ± 12.7	Total pethidine 24 h postoperatively (mg): 86.2 ± 16.7	NR	NR	NR	NR	NR	NR	Nausea and vomiting: 2 (10%) Bradycardia and hypotension: 1 (5%)	Nausea and vomiting: 5 (25%) Bradycardia and hypotension: 3 (15%)
Hefni et al., 2022 [16]	Time to first rescue analgesic (min): 21.6 ± 1.6 Total 24 h morphine consumption (mg): 3.8 ± 0.2	Time to first rescue analgesic (min): 11.5 ± 1.2 Total 24 h morphine consumption (mg): 16.9 ± 5.3	Fentanyl supplementation (μg): 57 ± 7	Fentanyl supplementation (μg): 58 ± 6	NR	NR	NR	NR	Nausea and/or vomiting: 0 (0) Bradycardia: 2 (3.7%) Hypotension: 3 (5.6%)	Nausea and/or vomiting: 8 (15.1) Bradycardia: 1 (1.89%) Hypotension: 2 (3.77%)
Jin et al., 2017 [17]	Time to first request pain medicine (h): 8.3 ± 6.6 Total tramadol consumption 48 h postoperatively (mg): 148.9 ± 74.8	Time to first request pain medicine (h): 6.4 ± 5.1 Total tramadol consumption (mg): 195.7 ± 66.2	Heart rate 0 min: 84.1 ± 6.9 30 min: 68.4 ± 8.3 60 min: 74.6 ± 10.7 120 min: 77.8 ± 9.8 Systolic blood pressure 0 min: 129.1 ± 11.6 30 min: 102.4 ± 11.8 60 min: 124.8 ± 12.2 120 min: 127.0 ± 12.5 Diastolic blood pressure 0 min: 81.4 ± 7.5 30 min: 64.9 ± 7.8 60 min: 75.1 ± 5.9 120 min: 78.0 ± 7.0	Heart rate 0 min: 83.5 ± 6.8 30 min: 79.1 ± 6.6 60 min: 80.1 ± 8.4 120 min: 80.3 ± 8.5 Systolic blood pressure 0 min: 127.8 ± 12.3 30 min: 120.1 ± 13.3 60 min: 131.9 ± 12.9 120 min: 133.2 ± 8.7 Diastolic blood pressure 0 min: 81.2 ± 7.1 30 min: 76.4 ± 7.4 60 min: 77.7 ± 7.6 120 min: 77.9 ± 6.7	VAS score 0 h: 2.5 ± 0.4 3 h: 2.2 ± 0.5 6 h: 2.4 ± 0.5 12 h: 2.5 ± 0.6 24 h: 2.4 ± 0.5 36 h: 2.3 ± 0.4 48 h: 2.3 ± 0.4	VAS score 0 h: 2.6 ± 0.4 3 h: 2.3 ± 0.6 6 h: 2.5 ± 0.6 12 h: 2.7 ± 0.7 24 h: 2.7 ± 0.6 36 h: 2.6 ± 0.5 48 h: 2.5 ± 0.6	NR	NR	Nausea: 4 (11.1%) Vomiting: 3 (8.3%) Pneumothorax: 1 (2.8%)	Nausea: 3 (8.3%) Vomiting: 2 (5.6%) Pneumothorax: 0
Kaur et al., 2017 [18]	Duration of analgesia (min): 469.6 ± 81.5 Total morphine consumption 24 h postoperatively (mg): 14.8 ± 2.4	Duration of analgesia (min): 298.2 ± 42.3 Total morphine consumption 24 h postoperatively (mg): 21.6 ± 3.1	NR	NR	VAS score 1 h: 2.3 ± 0.6 5 h: 2.4 ± 0.7	VAS score 1 h: 2.4 ± 0.7 5 h: 3.1 ± 0.8	NR	NR	Nausea and/or vomiting: 5 (16.6%)	Nausea and/or vomiting: 4 (13.3%)
Lakshmi et al., 2022 [19]	Total tramadol consumption 24 h postoperatively (mg/kg): 0.88 ± 0.707 Patient satisfaction score: Good (84%), Average (12%), Poor (4%)	Total tramadol consumption 24 h postoperatively (mg/kg): 2.84 ± 0 Patient satisfaction score: Good (28%), Average (56%), Poor (8%)	Heart rate: 79.90 ± 5.89 Onset of sensory blocks (min): 3.4 ± 0.70	Heart rate: 80.73 ± 6.79 Onset of sensory blocks (min): 4.76 ± 0.707	NR	NR	NR	NR	Hypotension: 4 (8.0%)	Hypotension: 0
Mohamed et al., 2014 [20]	Time to first request of analgesia (h): 8.16 ± 6.42 Total tramadol consumption 48 h postoperatively (mg): 150.19 ±76.98	Time to first request of analgesia (h): 6.48 ± 5.24 Total tramadol consumption 48 h postoperatively (mg): 194.44 ± 63.91	Heart rate 30 min: 69.8 ± 8.29 60 min: 74.43 ± 11.03 Systolic blood pressure 0 min: 130.00 ± 11.49 30 min: 101.33 ± 11.67 120 min: 127.20 ± 12.79	Heart rate 30 min: 79.33 ± 6.62 60 min: 80.40 ± 8.45 Systolic blood pressure 0 min: 126.57 ± 13.39 30 min: 118.60 ± 13.16 120 min: 134.0 ± 8.46	VAS score 1 h: 2.46 ± 0.6	VAS score 1 h: 2.51 ± 0.7	NR	NR	Nausea and/or vomiting: 4 (13.3%)	Nausea and/or vomiting: 6 (20.0%)
Mohta et al., 2015 [21]	Total morphine consumption 24 h postoperatively (mg): 2.4 ± 2.8 PCA morphine requirement 24 h postoperatively (mg): 1.5 ± 2.3 Fentanyl requirement (mcg): 54.6 ± 11.4 Time to mobilize (hour): 23.2 ± 4.0 Time to discharge (days): 5.2 ± 0.4	Total morphine consumption 24 h postoperatively (mg): 18.3 ± 13.5 PCA morphine requirement 24 h postoperatively (mg): 15.3 ± 13.1 Fentanyl requirement (mcg): 58.0 ± 10.3 Time to mobilize (hour): 43.4 ± 6.1 Time to discharge (days): 5.7 ± 0.5	NR	NR	NR	NR	NR	NR	Nausea and vomiting (PONV): 4 (26.7%) Hypotension: 13 (86.7%) Bradycardia: 4 (26.7%)	Nausea and vomiting (PONV): 11 (73.3%) Hypotension: 8 (53.3%) Bradycardia: 1 (6.67%)
Mostafa et al., 2018 [22]	Time to first request of analgesia (h): 7.8 ± 68 Total tramadol consumptions 48 h postoperatively (mg): 35 ± 5.3	Time to first request of analgesia (h): 3.25 ± 65 Total tramadol consumptions 48 h postoperatively (mg): 75 ± 7.2	HR 30 min: 66 ± 7 90 min: 75 ± 6	HR 30 min: 74 ± 3 60 min: 77 ± 5	NR	NR	NR	NR	NR	NR
Wang et al., 2021 [23]	PACU stay (min): 34.5 (29–48) Length of stay (days): 8 (6–18) Total flurbiprofen consumption 48 h postoperatively (mg): 100 (52–115)	PACU stay (min): 30 (24–45) Length of stay (days): 8 (6–15) Total flurbiprofen consumption 48 h postoperatively (mg): 150 (94–160)	Intraoperative propofol (mg): 461.7 ± 108.6 Intraoperative sufentanil (μg): 25 (20–30)	Intraoperative propofol (mg): 462.6 ± 112.1 Intraoperative sufentanil (μg): 30 (20–35)	VAS score at rest: 1 h: 0 (0–0) 6 h: 1 (1–1) 12 h: 1 (1–1) 24 h: 1 (1–1) 48 h: 1 (1–1) VAS score in movement: 1 h: 1 (1–1) 6 h 1 (1–1) 12 h: 2 (1–2) 24 h: 3 (2–3) 48 h: 3 (2–3)	VAS score at rest: 1 h: 0 (0–1) 6 h: 1 (1–1) 12 h: 1.5 (1–2) 24 h: 2 (1–2) 48 h: 1 (1–2) VAS score in movement: 1 h: 1 (1–1) 6 h: 1 (1–2) 12 h: 2.5 (2–3) 24 h: 3 (3–3) 48 h: 3 (2–3)	NR	NR	Nausea: 2 (6.65) Vomit: 0 Bradycardia: 0 Hypotension: 0	Nausea: 4 (13.3%) Vomit: 2 (6.6%) Bradycardia: 0 Hypotension: 0
Wu et al., 2022 [24]	PACU stay: 22.43 ± 3.98 Postoperative QoR-15 score: 109.5 (107–114) Patient satisfaction score:8.62 ± 0.59	PACU stay: 22.06 ± 3.76 Postoperative QoR-15 score: 107 (103–112) Patient satisfaction score: 8.28 ± 0.70	Total intraoperative propofol consumption (mg): 464.23 ± 28.21 Total intraoperative sufentanil consumption (μg): 21.22 ± 2.98 Total intraoperative remifentanil consumption (μg): 146.74 ± 14.99	Total intraoperative propofol consumption (mg): 470.27 ± 30.41 Total intraoperative sufentanil consumption (μg): 21.39 ± 3.07 Total intraoperative remifentanil consumption (μg): 151.54 ± 14.58	VAS score at rest: After 1 h: 0 (0–0.5) After 6 h: 0 (0–1) After 12 h: 1 (1–2) After 24 h: 2 (2–3) After 48 h: 2 (1–2) VAS score during movement: After 1 h: 1 (0–1) After 6 h: 1 (0–1) After 12 h: 2 (1–3) After 24 h: 3 (2–3) After 48 h: 2 (2–3)	VAS score at rest: After 1 h: 0 (0–0.5) After 6 h: 0 (0–1) After 12 h: 1 (1–2) After 24 h: 2 (2–3) After 48 h: 2 (1–2) VAS score during movement: After 1 h: 1 (0–1) After 6 h: 1 (0–1) After 12 h: 2 (1–3) After 24 h: 3 (2–3) After 48 h: 2 (2–3)	NR	NR	Nausea and vomiting (PONV): After 24 h: 6 (16.2%) After 48 h: 5 (13.5%) Bradycardia: 1 (2.7%) Dizziness: 3 (8.1%) Delirium: 0	Nausea and vomiting (PONV): After 24 h: 11 (30.6%) After 48 h: 8 (22.25%) Bradycardia: 0 Dizziness: 4 (11.1%) Delirium: 0
Salem et al., 2019 [25]	Time to first request of analgesia (h): 15 (4–20) Total Morphine consumption 24 h postoperatively (mg): 1.06± 2.33	Time to first request of analgesia (h): 6 (4–14) Total Morphine consumption 24 h postoperatively (mg): 3.28 ± 4.67	NR	NR	VAS score 2 h: 1 (0–2) 6 h: 4 (2–4) 12 h: 5 (3–5)	VAS score 2 h: 2 (0–3) 6 h: 5 (3–7) 12 h: 7 (4–8)	Serum cortisol (μg/dL): baseline: 16.1 ± 1.4 postop: 8.6 ± 1.1	Serum cortisol (μg/dL): baseline: 16.3 ± 1.4 postop: 14.4 ± 1.8	Nausea and/or vomiting: 5 (16.6%)	Nausea and/or vomiting: 30 (100%)
Wan et al., 2022 [26]	Duration of PCA (h): 4.7 ± 1.5 Total sufentanil consumption via PCA in 48 h after operation (ml): 32.5 ± 5.4 PCA pressing times in 48 h after operation (n): 11.4 ± 1.7	Duration of PCA (h): 6.3 ± 2.4 Total sufentanil consumption via PCA in 48 h after operation (ml): 45.4 ± 8.3 PCA pressing times in 48 h after operation (n): 14.3 ± 2.5	NR	NR	NR	NR	NR	NR	Nausea and vomiting (PONV): 2 (5.0%) Cardiac arrhythmia: 0 Respiratory depression: 0 Bradycardia: 1 (2.5%) Hypotension: 1 (2.5%)	Nausea and vomiting (PONV): 5 (12.5%) Cardiac arrhythmia: 0 Respiratory depression: 1 (2.5%) Bradycardia: 1 (2.5%) Hypotension: 2 (5.0%)
Wang et al., 2022 [27]	Total sufentanil consumption 48 h postoperatively (μg): 23.3 ± 10.0	Total sufentanil consumption 48 h postoperatively (μg): 33.8 ± 13.8	NR	NR	NR	NR	NR	NR	Nausea and/or vomiting: 4 (13.3%)	Nausea and/or vomiting: 8 (26.7%)
Elshal et al., 2021 [28]	Patients required rescue analgesia in the 1st 24 h postoperative: 11 (54%) Total morphine consumption in 24 h postoperative (mg): 3 (0–3) Time to first request of rescue analgesia (h): 6 (6–12)	Patients required rescue analgesia in the 1st 24 h postoperative: 15 (71.4%) Total morphine consumption in 24 h postoperative (mg): 3 (0–6) Time to first request of rescue analgesia (h): 4 (3–6)	Total intraoperative fentanyl consumption (μg): 153.33 ± 23.09	Total intraoperative fentanyl consumption (μg): 169.05 ± 31.88	NR	NR	NR	NR	Intraoperative hypothermia: 6 (28.6%) Nausea and vomiting (PONV): 7 (33.3%) Block-related complication: 0	Intraoperative hypothermia: 7 (33.3%) Nausea and vomiting (PONV): 5 (23.8%) Block-related complication: 0
Xu et al., 2018 [29]	Patient satisfaction (5-point Likert scale): 1: 0 2: 0 3: 1 (3.3%) 4: 9 (30%) 5: 20 (66.7%)	Patient satisfaction (5-point Likert scale): 1: 0 2: 0 3: 7 (23.3%) 4: 17 (56.7%) 5: 6 (20.0%)	Total intraoperative fentanyl consumption (μg): 561.7 ± 145.4	Total intraoperative fentanyl consumption (μg): 583.3 ± 124.1	NR	NR	NR	NR	Nausea and vomiting (PONV): 2 (6.7%) Bradycardia: 2 (6.7%) Hypotension: 1 (3.3%) Respiratory depression: 0	Nausea and vomiting (PONV): 3 (10.0%) Bradycardia: 0 Hypotension: 0 Respiratory depression: 0
Kassim et al., 2021 [32]	Time to first request for analgesic (h): 8.90 ± 2.47 Total nalbuphine consumption 24 h postoperatively (mg/kg): 0.15 ± 0.0	Time to first request for analgesic (h): 4.4 ± 1.05 Total nalbuphine consumption 24 h postoperatively (mg/kg): 0.24 ± 0.08	NR	NR	VAS score 0 h: 0.45 ± 0.51 2 h: 0.75 ± 0.4 6 h: 2.45 ± 0.51	VAS score 0 h: 0.90 ± 0.31 2 h: 1.75 ± 0.4 6 h: 3.20 ± 0.6	NR	NR	Nausea and/or vomiting: 2 (10.0%)	Nausea and/or vomiting: 4 (20%)

**Table 3:** Summary of findings: dexmedetomidine compared with control group.

Outcomes	Effect in Intervention Group	Participants		Studies (n)	Quality of Evidence (GRADE)
		Dexmedetomidine	Control		
Total 24 h morphine consumption	SMD = −1.99 [95% CI −3.01 to −0.98]	147	147	5	Low ^1,2^
Total 48 h postoperative tramadol consumption	SMD = −2.27 [95% CI −4.18 to −0.35]	82	82	3	Low ^2,3^
Total 48 h postoperative sulfentanil consumption	SMD = −1.34 [95% CI −2.29 to −0.40]	70	70	2	Moderate ^2^
First rescue morphine analgesia	SMD = 2.98 [95% CI 0.01 to 5.95]	113	113	3	Low ^2,3^
First rescue tramadol analgesia	SMD = 0.24 [95% CI −0.06 to 0.55]	82	82	3	Moderate ^3^
Postoperative mean VAS score	SMD = −0.40 [95% CI −0.71 to −0.10]	188	188	4	Low ^1,2^
Adverse events: postoperative nausea and vomiting	RR 0.54 [95% CI 0.37 to 0.79]	485	484	16	Moderate ^1^
Adverse events: bradycardia	RR 2.56 [95% CI 1.11 to 5.88]	245	244	7	Moderate ^1^
Adverse events: hypotension	RR 1.93 [95% CI 0.0.93 to 4.00]	258	258	7	Low ^1,2^

GRADE certainty ratings were as follows—high: the authors have a lot of confidence that the true effect is similar to the estimated effect; moderate: the authors believe that the true effect is probably close to the estimated effect; low: the true effect might be markedly different from the estimated effect; and very low: the true effect is probably markedly different from the estimated effect. ^1^ Downgraded to 1 level for risk of bias. ^2^ Downgraded to 1 level for inconsistency. ^3^ Downgraded to 1 level for imprecision.

## Data Availability

Data are available in a publicly accessible repository.

## Supplementary Materials

The following supporting information can be downloaded at: https://www.mdpi.com/article/10.3390/jcm13113166/s1, Table S1. Literature Search; Table S2. Quality assessment of ROB.2 for RCTs studies; Table S3. GRADE Assessment of Evidence; Figure S1. Funnel plot of meta-analysis for total opioid consumption; Figure S2. Funnel plot of meta-analysis for time to first rescue analgesic; Figure S3. Funnel plot of meta-analysis for VAS score; Figure S4. Funnel plot of meta-analysis for any adverse events: A) PONV; B) bradycardia and hypotension.
