# Supplementary material for: Dexmedetomidine as an Adjuvant to Nerve Block for Cancer Surgery: A Systematic Review and Meta-Analysis
Source: J Clin Med. 2024 May 28;13(11):3166. doi: 10.3390/jcm13113166 (PMC11172819; doi:10.3390/jcm13113166)
Supplement: Supplementary file 1 [file jcm-13-03166-s001.zip › jcm-3025873-supplementary.pdf]

SUPPLEMENTARY MATERIALS

Table S1. Literature Search

- Keywords

#1 (Regional anaesthesia) OR (Nerve block) OR (Truncal nerve block)
#2 (Dexmedetomidine)
#3 (Cancer) OR (Cancer surgery)
Search Results

Database	Keywords	Search Result	Search-time
MEDLINE	#1 AND #2 AND #3	84	May 17, 2024
ScienceDirect	#1 AND #2 AND #3	1257	May 17, 2024
Cochrane Library	#1 AND #2 AND #3	68	May 17, 2024
Springer	#1 AND #2 AND #3	723	May 17, 2024
Scopus	#1 AND #2 AND #3	175	May 17, 2024
MedRxiv	-	14	May 17, 2024

Table S2. Quality assessment of ROB.2 for RCTs studies

Authors	Domain					Overall	Result
	D1	D2	D3	D4	D5		
Bakr et al.,2018							Low risk
Fattah et al.,2019							Some concerns
Hawas et al.,2021							Low risk
Hefni et al.,2022							Low risk
Kaur et al.,2017							Low risk
Mohamed et al.,2014							Low risk
Mostafa et al.,2018							Low risk
Wang et al., 2021							Low risk
Jin et al., 2017							Low risk
Abdelzaam et al., 2020							Low risk
Hassan et al., 2023							Some concerns
Mohta et al., 2015							Low risk
Wu et al., 2022							Low risk
Lakshmi et al., 2022							Low risk

Domains

D1 : bias arising from the randomization process
D2 : bias due to deviations from intended interventions
D3 : bias due to missing outcome data
D4 : bias in measurement of the outcome
D5 : bias in selection of the reported result

Low risk
some concerns
high risk

Continued. Quality assessment of ROB.2 for RCTs studies

Authors	Domain					Overall	Result
	D1	D2	D3	D4	D5		
Salem et al.,2019							Low risk
Wang et al.,2022							Low risk
Wan et al., 2022							Low risk
Elshal et al., 2021							Low risk
Xu et al., 2018							Low risk
Kassim et al.,2021							Some concerns

Domains

D1 : bias arising from the randomization process
D2 : bias due to deviations from intended interventions
D3 : bias due to missing outcome data
D4 : bias in measurement of the outcome
D5 : bias in selection of the reported result

Low risk
some concerns
high risk

Table S3. GRADE Assessment of Evidence

Certainty assessment							No of patients		Certainty
No of studies	Study design	Risk of bias	Inconsistency	Indirectness	Imprecision	Other considerations	Dexmedeto midine	Standard Care	
Total 24-hours morphine consumption									
5	RCT	serious[a]	Serious[b]	not serious	not serious	none	147	147	⨁⨁◯◯ LOW
Total 48-hours postoperative tramadol consumption									
3	RCT	not serious	Serious[b]	not serious	Serious[c]	none	82	82	⨁⨁◯◯ LOW
Total 48-hours postoperative sufentanil consumption									
2	RCT	not serious	Serious[b]	not serious	not serious	None	70	70	⨁⨁⨁◯ MODERATE
First rescue morphine analgesia									
3	RCT	not serious	Serious[b]	not serious	Serious[c]	none	113	113	⨁⨁◯◯ LOW
First rescue tramadol analgesia									
3	RCT	not serious	not serious	not serious	Serious[c]	None	82	82	⨁⨁⨁◯ MODERATE
Post-operative Mean VAS Score									
4	RCT	Serious[a]	Serious[b]	not serious	not serious	none	188	188	⨁⨁◯◯ LOW
Adverse events : postoperative nausea and vomiting									
16	RCT	serious[a]	not serious	not serious	not serious	none	485	484	⨁⨁⨁◯ MODERATE
Adverse events : bradycardia									
7	RCT	serious[a]	not serious	not serious	not serious	none	245	244	⨁⨁⨁◯ MODERATE
Adverse events : hypotension									
7	RCT	serious[a]	Serious[b]	not serious	not serious	None	258	258	⨁⨁◯◯ LOW

Explanations

a. 1 RCTs are Open Label; b. $I^2 > 30\%$ and $P<0.05$; c. There are extreme confidence intervals of effect estimates

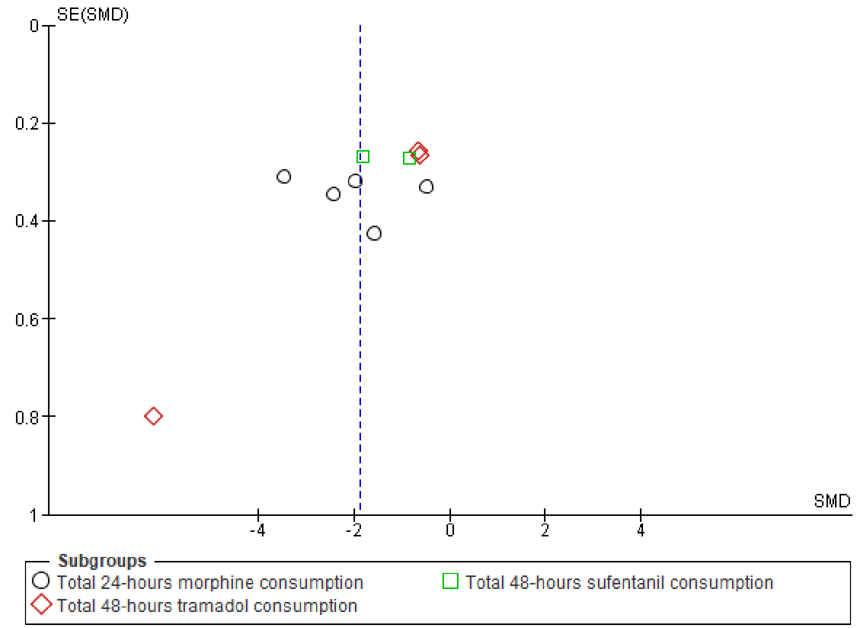

Figure S1. Funnel plot of meta-analysis for total opioid consumption.

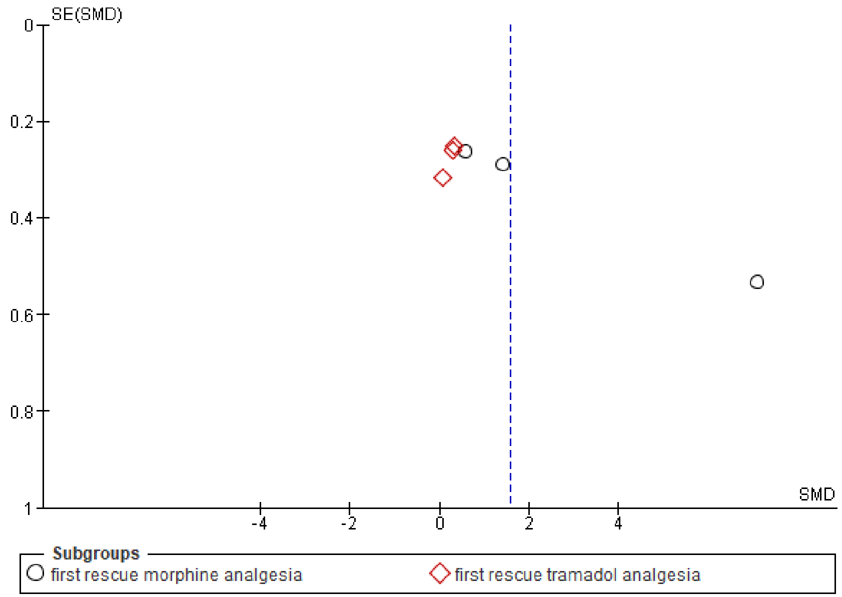

Figure S2. Funnel plot of meta-analysis for time to first rescue analgesic.

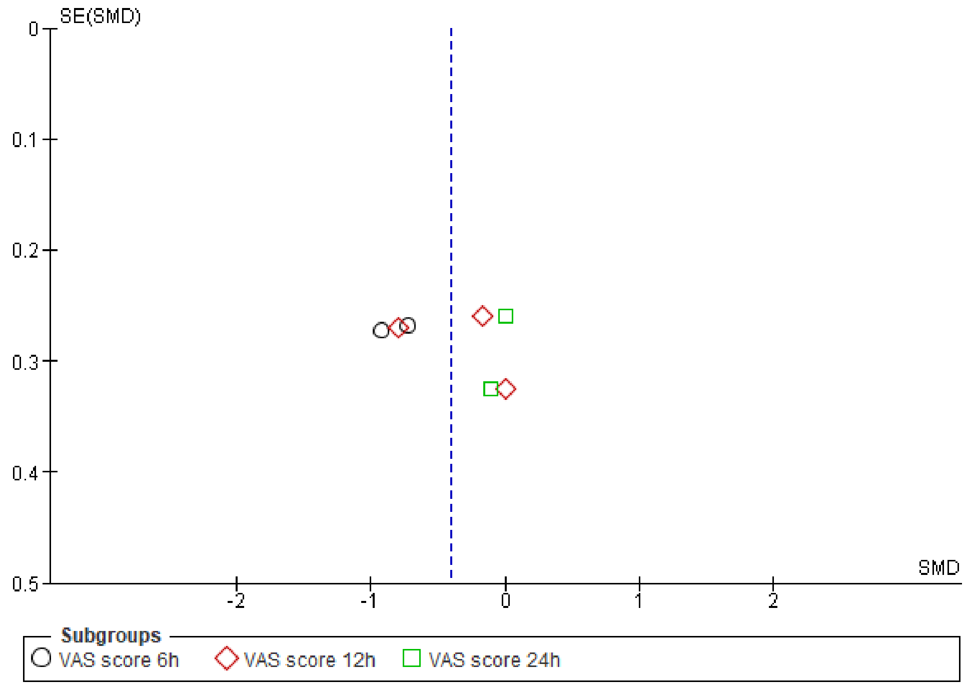

Figure S3. Funnel plot of meta-analysis for VAS score.

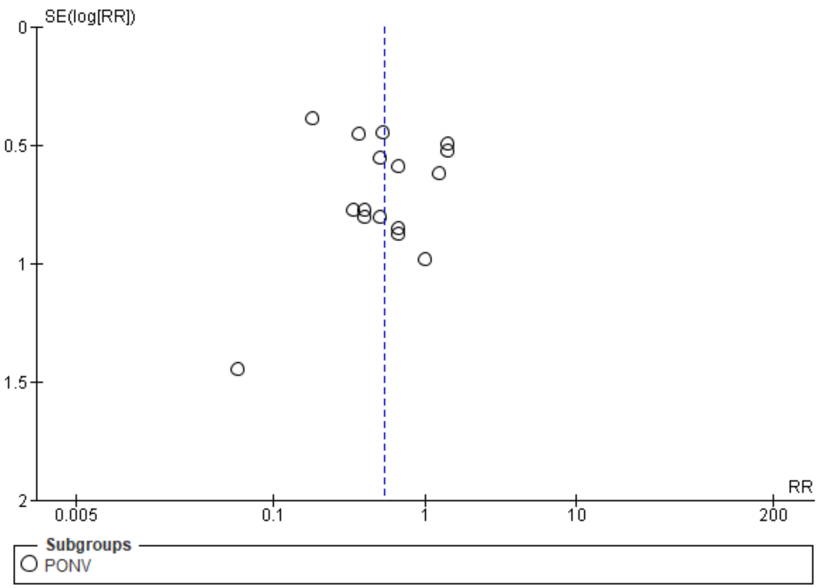

A

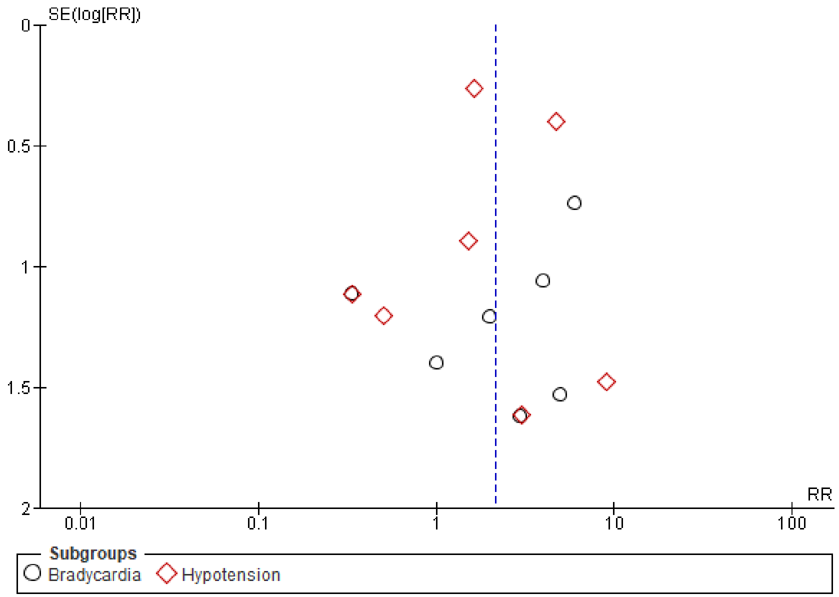

B

Figure S4. Funnel plot of meta-analysis for any adverse events: A) PONV; B) bradycardia and hypotension.
